# Research priorities in trans health: a Delphi-study

**DOI:** 10.3389/fpubh.2024.1307531

**Published:** 2024-04-12

**Authors:** Lucie Jurek, Marie De la Chenelière, Marion Lapoirie, Paul Neuville

**Affiliations:** ^1^Child and Adolescent Psychiatry Department, Centre Hospitalier Le Vinatier, Bron, France; ^2^RESHAPE, Research on Healthcare Performance, Claude Bernard Lyon 1 University, Lyon, France; ^3^Maison Dispersée de Santé, Lille, France; ^4^Service de Médecine de la Reproduction, Hôpital Femme-mère-enfant, Hospices Civils de Lyon, Bron, France; ^5^Urology Department, Hôpital Lyon Sud, Hospices Civils de Lyon, Lyon, France; ^6^Claude Bernard University Lyon 1, Lyon, France

**Keywords:** Delphi-study, research priorities, trans health, transgender, thematic analysis

## Abstract

**Purpose:**

Progress has been made in understanding trans health needs, but research priorities are often set by policy or healthcare professionals without trans input, which may not reflect public needs. Our study sought to identify trans health research priorities in France from both researchers and the trans community.

**Methods:**

Expert stakeholders (health and social sciences professionals, trans individuals, and their families) answered a three-round Delphi survey on trans health research priorities. The first round involved an open-ended questionnaire, analyzed qualitatively. In the second round, participants ranked research propositions from round one using a Likert scale. The study’s second phase involved a two-hour workshop with experts and trans individuals.

**Results:**

53 participants (32% trans individuals/relatives, 60% health professionals) contributed 217 responses to open-ended questions, leading to 44 research priorities. After the two voting rounds, a total of five proposals reached a strong consensus cut-off and were considered as the main research priorities: evaluation of the effect of puberty blocker use in trans children and adolescents (95%), evaluation of the effect of supporting trans children and adolescents (92%), study of the support systems available for trans youth and their parents (86%), persistence of trans identity around puberty (prevalence, persistent persons characteristics) (86%), and needs assessment survey of the support for adolescents and their families (83%). Thirteen other proposals were considered moderate priorities.

**Conclusion:**

The main consensus in our French study concerned research on trans-youth care and support needs. Our results may guide further trans-health research that meets the public’s needs and desires.

## Introduction

Over the past two decades, there have been significant advances in understanding the health needs of trans-communities and how to approach their care (1). Despite this progress, significant gaps in knowledge still exist in almost all aspects of trans-health. Most reported studies had short durations, small sample sizes, or a lack of control or comparison populations (2, 3). Research on specific health outcomes for trans individuals is also limited, with only a few small-scale studies valuing their voices in the research process.

Research priorities are typically established by public policy, healthcare professionals, guideline developers, researchers, and research funders, with little public input. As a result, research priorities may not align with public needs and preferences (4).

The importance of involving the trans community in conducting research on trans health has been advocated and should take place throughout every stage of the research process to identify and focus on research areas that are most meaningful and impactful to the trans community (5). Their involvement appears to be even more essential for defining research priorities to avoid ignoring community needs and exploring specific topics regarding health and well-being that might not otherwise be identified (6, 7). Concrete advances in trans-health research may thereby be possible by identifying the unmet medical needs of this population (8). Attempts to define research priorities in trans health have been previously reported, mostly based on literature reviews (2, 3, 9), and by expert groups that do not always involve the trans community (10). Research on the transgender population has been marred by flawed practices, highlighting the need for greater involvement of the trans community (11). Conducting research with a foundation in meaningful collaboration with the trans community is now a crucial guideline (12) for undertaking transgender health research. This approach ensures a more nuanced and respectful exploration of the diverse experiences within the trans community, ultimately contributing to the development of more accurate and relevant findings.

Moreover, the Delphi method stands out as a widely employed tool for achieving expert consensus (13). This method treats each participant as an expert, and its application to a panel comprising both trans care providers and individuals within the transgender community holds the potential to yield novel insights into research priorities.

In this article we aim to determine research priorities in trans health in France using a Delphi study method to retrieve opinions from researchers and the trans community.

## Methods

### Overview

In this article, we will use the broad definition of “trans” to include any person whose gender identity differs from societal expectations of their assigned sex at birth. This includes transgender, non-binary, and other gender-diverse people (14). We use the term “trans-health” to address health issues for trans people.

This study consisted in a 3-round modified Delphi study (13, 15) followed by a 2 h workshop during the French Trans-Health Congress (October 6th–7th, 2022), in which participants discussed the results of the first phase of the study. The Delphi method is an iterative *a priori* process in which a group of expert stakeholders come to a structured consensus view of a particular topic through a number of rounds with controlled feedback (16). Delphi studies have been successfully conducted to establish research priorities for numerous different topic areas, including cancer caregiver intervention (17), or public health research to address health inequalities (18).

### Population selection

Participants were contacted using the Trans-Santé France (TSF)/French Professional Association for Transgender Health (FPATH) mailing list. TSF/FPATH is a French association that works to improve trans-health in France. The association is composed of health professionals from diverse medical specialties (*N* = 108 [69%], at the time of the study), professionals from social sciences (*N* = 6 [4%]), and transgender individuals and their families (*N* = 42 [27%]). The association’s members are all over 18. Our research was designed to ensure the highest level of anonymity for the members of the association. To achieve this, we opted to group the categories of transgender individuals and their relatives (notably, at the time of the study, only two relatives of transgender individuals were actively involved in the association). Additionally, we refrained from collecting and disclosing the age of participants, further safeguarding their privacy.

### Ethic statements

French law “loi Jardé” regarding research involving human subjects does not require an ethics committee for studies in the field of health sociology or for survey on health practices (19). Our study falls within this context. This study was conducted in accordance with the Helsinki Declaration. All participants contacted for the study received clear, fair, and informed information about the study’s content. Their written consent was obtained prior to their participation in the questionnaire. Additionally, the data was stored on a secure server at the University of Lyon 1.

### Delphi round 1: open-ended questionnaire

In June 2022, the first survey was sent to the TSF/FPATH mailing list (*N* = 156). The survey comprised a single open-ended question: «In your opinion, what are the 5 issues/themes that research should prioritize in the field of trans health?».

Responses to the open-ended questions were analyzed using two different approaches. First, a content analysis method was used to determine the representation of the different research areas within the proposals (20). The first analysis was performed by one researcher and then verified by a second. Second, thematic qualitative analysis was conducted independently by two researchers (21). The results of the open-ended questions were coded line-by-line in an inductive manner. The codes were grouped into themes that corresponded to research priorities. In cases of disagreement between the two researchers, the themes were discussed with a third researcher until a consensus was reached.

### Delphi round 2 and 3

In round 2, a second survey was sent in August 2022 to participants of the first round. Participants were asked to evaluate the priority of each research proposition retrieved from round 1 using a 5-point Likert survey (“High priority,” “Priority,” “Neutral,” “Low priority,” “Not a priority”).

In round 3, the frequency and percentage of agreement for each proposal were calculated and fed back to the participants for an appreciation of the general response of all the panels. After receiving the feedback, participants were asked to vote again in September 2022 using the same Likert scale on the same items.

### Congress discussion

The main results of the 3 phases Delphi were presented and discussed during the 2nd TSF/FPATH congress In Lyon (October 6th–7th, 2022). Two facilitators led the discussion (PN, LJ). Notes were taken by a third person during the session. These notes were analyzed using a thematic analysis method and the code book from round 1. New codes emerging from the data could be added to the code book.

### Analysis and consensus

Statistical analyses were performed using R Studio with the Likert package. The Likert categories were grouped as follows: “High priority” and “Priority” were grouped as “Priority,” “Low priority” and “Not a priority” were grouped as “Non-priority.” The category “neutral” did not change.

Delphi studies use variable definitions and thresholds to determine opinion consensus (15). Our study used a percentage equal to or higher than 80% to define strong consensus. Proposals between 70 and 80% were considered moderate consensus. The Delphi was set *a priori* to run over three rounds unless no research priority reached consensus.

## Results

### Population

A total of 156 individuals were contacted by mail. Fifty-three participated in the first and second rounds of the study, and 42 in the third round. A third (32%) of the respondents were trans individuals or their relatives, while the others were mainly health professionals (60%) (Table 1).

**Table 1:** Population characteristics.

Participants (*n* = 53)	*N* (%)
A trans individual or a relative	17 (32%)
A health professional Psychiatrist Reproduction biologist Surgeon Endocrinologist General Practitioner Gynecologist Nurse and caregivers Psychologist Sexologist	32 (60%) 6 3 7 3 4 3 4 1 1
A social sciences professional	4 (8%)

N, number.

### Delphi round 1

**Figure 1:**
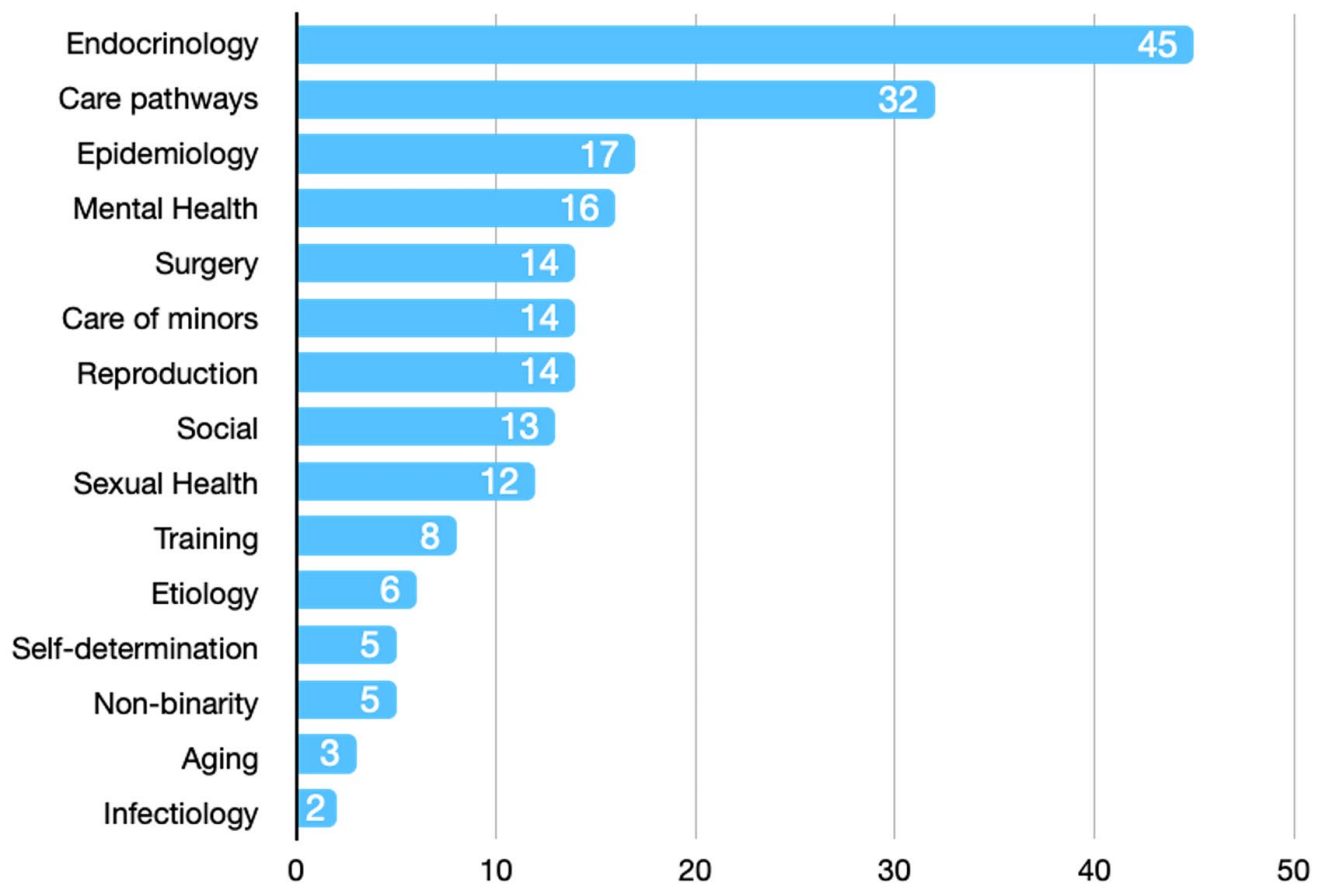
Content analysis. Absolute number of proposal by research field.

A total of 217 responses to open-ended questions were analyzed. Endocrinology and care pathways were the two main components retrieved from the content analysis (Figure 1). Forty-four research priorities were retrieved from the 217 open-ended proposals.

### Delphi round 2 and 3

**Figure 2:**
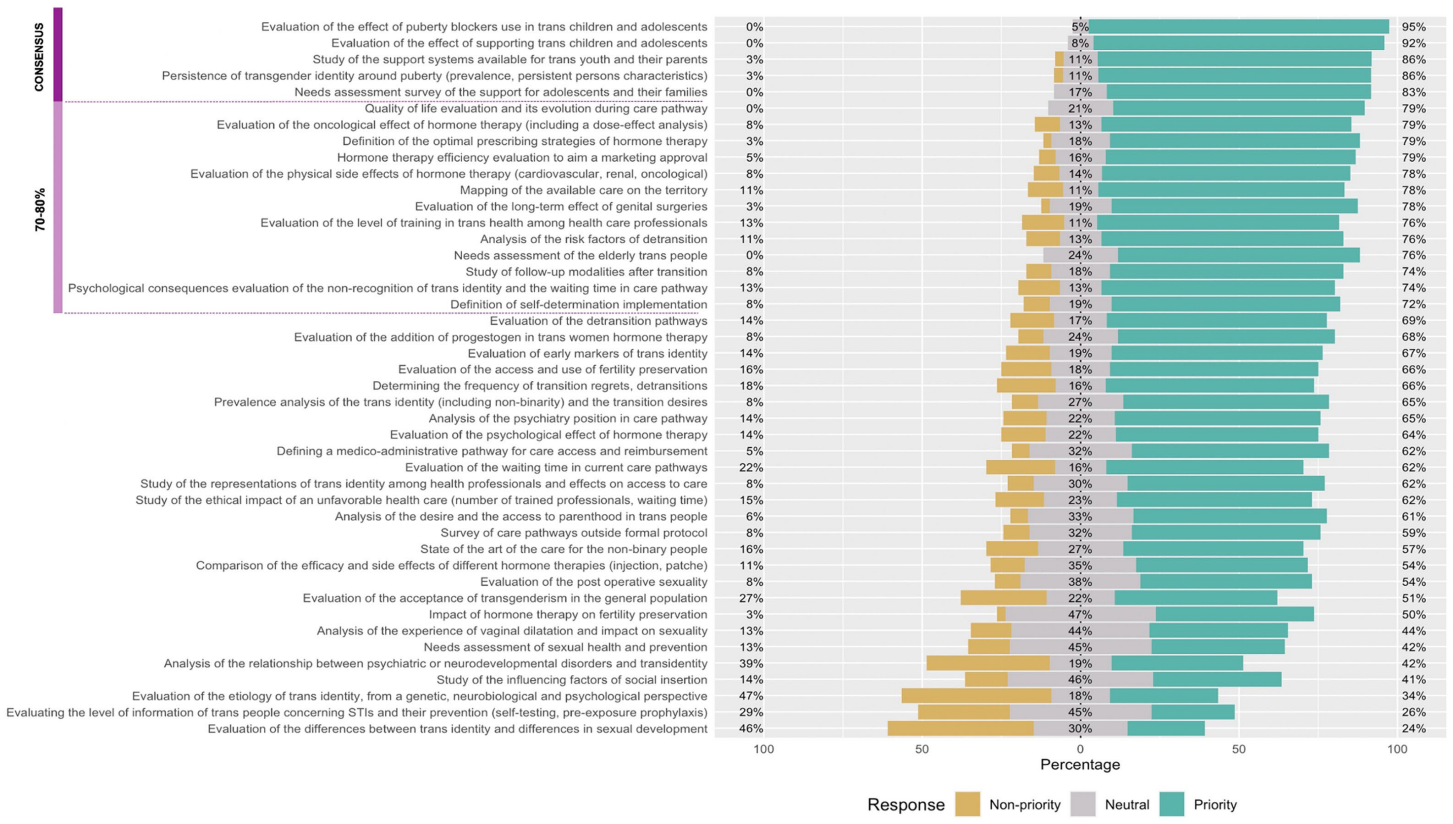
Research priorities assessment. STI, sexually transmitted infections.

The results of the two Delphi rounds are shown in Figure 2. A total of five proposals reached an 80% strong consensus cut-off and were considered as the main research priorities: evaluation of the effect of puberty blocker use in trans children and adolescents (95%), evaluation of the effect of supporting trans children and adolescents (92%), study of the support systems available for trans youth and their parents (86%), persistence of trans identity around puberty (prevalence, persistent persons characteristics) (86%), and needs assessment survey of the support for adolescents and their families (83%).

Thirteen proposals were considered moderate consensus. Four of them were related to endocrinology: evaluation of the oncological effect of hormone therapy (including a dose-effect analysis) (79%), definition of the optimal prescribing strategies of hormone therapy (79%), hormone therapy efficiency evaluation to aim a marketing approval (79%), evaluation of the physical side effects of hormone therapy (cardiovascular, renal, oncological) (78%).

Five related to long term outcomes such as quality of life evaluation and its evolution during care pathway (79%), needs assessment of the older adults trans people (76%), study of follow-up modalities after transition (74%), analysis of the risk factors of detransition (76%), evaluation of the long-term effect of genital surgeries (78%).

Two proposals concerned health resources with the idea of mapping the available care on the territory (78%) and the evaluation of the level of training in trans health among healthcare professionals (76%).

Psychological consequences evaluation of the non-recognition of trans identity and the waiting time in care pathway also reached a moderate consensus (74%), as did the definition of self-determination implementation (72%).

Different themes such as sexual health, infectiology, or fertility/parenting were represented in the proposals that did not reach a consensus (Figure 2).

Research on etiology or on the link between trans identity and genital development disorders or psychiatric disorders was largely voted as “non-priority.”

### Congress discussion

Around thirty people participated in the specific session of the congress. No new themes emerged during the Congress. The participants agreed with the consensus proposals. Participants could discuss the proposals that received the most “non-priority” votes. Some health professionals’ participants were surprised to see little interest in the co-occurrence of psychiatric disorders, which is frequently described in the literature. The issue of the stigma attached to this association is discussed. Abundant literature on this topic has also been described as influencing non-prioritization.

## Discussion

### Main results and comparison to scientific literature

Our study aimed to evaluate the research priorities in trans health from the perspectives of French health professionals and service users. Among the 44 research proposals, five reached a strong consensus (>80%), and 13 reached a moderate consensus (70–80%).

The 5 top research priorities concerned research on trans minors and their parents. Research to evaluate the efficacy of care, such as puberty blockers, as well as the assessment of families’ support needs and existing support systems, has been proposed. These proposals are consistent with the available literature on research priorities in this field, which regrets the lack of well-designed studies on specific care for trans youth (3). Research priority given to trans youth in the present study may also be a consequence of the high level of unmet healthcare needs of this population (22). The proposal of a follow-up study to determine the persistence of trans identity around puberty also retrieved a high number of votes. Participants exposed the need for robust estimates to move forward with discussions regarding trans youth trajectories, aligning with the need for updated data and a more diverse population previously reported (3).

This importance given to a better knowledge of the trans youth path is possibly a response to trans youth care being intensively in the spotlight (ban of gender-affirming care for minors in Texas (23), limitations to medical care in Sweden and the United Kingdom) (24).

Moderate consensus proposals focused on hormone therapy and healthcare modalities, such as access, needs, and follow-up. The research priority given to hormone therapy has been consistently highlighted in a recent review (9). The importance of improving knowledge in this field emerged in the first round of the Delphi process, as endocrinology was the most frequently mentioned topic resulting from the open-ended questions. The research field of hormone therapy encompasses various areas, including the assessment of oncological risks, which was previously identified as a research priority (2). An augmented oncological risk associated with hormonotherapy appears to be low in the current literature, acknowledging the fact that more powerful data are still necessary in the field (25). Still, the impact on health can be important, as recently outlined by the association between meningiomas and cyproterone acetate (26).

Evaluating the effectiveness of hormone therapy to obtain marketing approval is another important aspect, yet very specific within the French health reimbursement system. This wide range of research proposals regarding hormonotherapy may be a consequence of the limited number of dedicated studies that were conducted during the beginning of the use of hormone therapy in the trans population, in the context of poor access to healthcare and a high frequency of self-prescription (27).

The initial open-ended question of the present study asked for the five issues/themes that research should prioritize in the field of trans health. Yet a broad question, this formulation did not allow the collection of any responses regarding research modalities, even if longitudinal evaluation has been reported herein as a priority with moderate consensus. Research modalities are expected to be defined in further steps to examine the best methodology for answering a prioritized research question. However, we acknowledge the need for prospective data collected by well-designed, long-term studies or for translational research previously underlined by some authors (2, 3, 9).

One important criterion regarding the decision to prioritize research would be to consider whether this study will benefit individual care (28). The absence of priority given to research on representation or acceptance may be driven by the absence of direct benefits in the care pathway, as stated by some participants in our study. Another important criterion, from a researcher’s point of view, is the gap to fill in the literature. The rich literature on sexually transmitted infections may explain the lack of priority given to this theme (29, 30).

Surprisingly, our study did not retrieve any research priority focusing on trans-specific outcomes, although this has been described in recent reviews (2, 3, 9). Similarly, no proposal regarding the impact of social determinants of health or health-promoting factors and resiliency was retained, although some studies found it to be the main priority in their qualitative study on research priorities in trans health (6, 31).

### Strengths and limitations

Our study has several strengths. This is the first study to assess research priorities in trans-health using a rigorous Delphi methodology. Our population was composed of one-third of trans individuals and their families, as we wanted our findings to match trans community needs. We conducted a congress discussion as a triangulation method to validate our results and provide a deeper understanding.

However, we must acknowledge that the trans individuals recruited through the TSF association and during TSF scientific committees are persons with a certain degree of awareness and endorsement of a medical approach and may not entirely share the same priorities with a larger trans community. Another limitation is the absence of a trans researcher in the project design (32). Our population also lacked nurses, social workers, and dermatologists. These limitations may result from selection bias, as we used the mailing list of only one professional association for recruitment. Yet, TSF is currently the main association of health professionals engaged in trans-care in France, and its statutes align with the main principles of trans-health-related research (12). Broader participation may have changed some of our results, which should be interpreted with regard to this information.

We did not perform sub-group analysis, which could allow us to compare the priorities between the trans community and health professionals, but as described above, we wanted to extract research priorities that reflect both professionals’ and individuals’ experiences. Although the response rate in our study was relatively low, it is noteworthy that the recruited population closely resembled the overall demographics of TSF members, maintaining an overarching ratio of 1/3 trans individuals to 2/3 health professionals. Despite the challenges in response rates, our sample achieved a valuable diversity in terms of professions and representation of transgender individuals.

## Conclusion

Our study identified research priorities in trans-health from the perspectives of professional and service users. A high consensus on priority was reached regarding the need for research on trans-youth care efficacy and support. Further steps are required to ensure the translation of priority research themes into a co-constructed, well-structured, ethical research methodology.

## Data availability statement

The raw data supporting the conclusions of this article will be made available by the authors, without undue reservation.

## Ethics statement

Ethical approval was not required for the studies involving humans because French law “loi Jardé” regarding research involving human subjects does not require an ethics committee for studies in the field of health sociology or for survey on health practices. Our study falls within this context. The studies were conducted in accordance with the local legislation and institutional requirements. The participants provided their written informed consent to participate in this study.

## Author contributions

LJ: Writing – review & editing, Writing – original draft, Visualization, Methodology, Investigation, Formal analysis, Data curation, Conceptualization. MC: Writing – review & editing, Writing – original draft. ML: Writing – review & editing, Writing – original draft. PN: Writing – review & editing, Writing – original draft, Methodology, Investigation, Formal analysis, Data curation, Conceptualization.
